# ARE OBESITY AND ADENOMA DEVELOPMENT ASSOCIATED AS COLORECTAL CANCER PRECURSORS?

**DOI:** 10.1590/0102-672020190001e1500

**Published:** 2020-07-08

**Authors:** Bianca Astrogildo de FREITAS, Carlos Alberto Tomatis LOTH, Gustavo Lazaroto SWAROWSKY, Graziela Morais LOURENÇO, Lucio Sarubbi FILLMANN, Henrique Sarubbi FILLMANN, Maria Luiza SANTOS, Alexandre Vontobel PADOIN

**Affiliations:** 1 Pontifical University do Rio Grande do Sul, School of Medicine, Porto Alegre, RS, Brazil; 2 Faculty of Medicine, Federal University of Rio Grande, Rio Grande, RS, Brazil

**Keywords:** Adenoma, Colonoscopy, Obesity, Colorectal neoplasms, Adenoma, Colonoscopia, Obesidade, Neoplasias colorretais

## Abstract

***Background*:**

One of the most important concerns on health is the increased rates of obesity in population and the speed in which this number is increasing. This number translates a serious public health problem, since it also increases the risk of several other diseases associated with obesity resulting in significant morbidity and mortality. Among them, it seems to be connected to several neoplasms, such as colorectal carcinoma.

***Aim*:**

To evaluate the impact of obesity as a risk factor for colorectal carcinoma through the detection of adenoma, and to discuss the mechanisms that could establish a link between obesity and neoplasm.

***Methods*:**

Patients who underwent colonoscopy were included. Personal and anthropometric data, clinical history, and results of the tests were analyzed in order to verify the correlation of BMI and the presence of adenomatous polyps.

***Results*:**

A total of 142 patients were studied, which a mean age of 62 years. Of the patients, 74 (52.1%) were men and 68 (47.9%) were. Obesity was identified in 16.2% of the patients. Polyps were found in 61 (42.9%), mostly smaller than 1 cm. Obese individuals were 1.56 times more likely to present colorectal adenoma than patients with normal weight.

***Conclusion*:**

This study, although showing the greater presence of colorectal adenomas in obese individuals, did not show a significant difference in the occurrence of pre-malignant lesions.

## RESUMO

***Racional*:**

Uma das grandes preocupações no âmbito da saúde é o crescente índice de obesidade na população e a velocidade com que esse número vem aumentando. Ele constitui grave problema de saúde pública, uma vez que aumenta também o risco de inúmeras doenças associadas à obesidade e que resultam em morbimortalidade significativa, como o câncer colorretal.

***Objetivo*:**

Avaliar o impacto da obesidade como fator de risco para câncer colorretal, através da detecção de adenomas colorretais, e discutir os mecanismos que podem estabelecer uma ligação entre esta neoplasia e a obesidade.

***Métodos*:**

Foram incluídos pacientes submetidos à colonoscopia. Dados pessoais e antropométricos, antecedentes clínicos e laudos dos exames foram analisados, a fim de verificar a correlação do IMC e a presença de pólipos adenomatosos.

***Resultados*:**

Foram estudados 142 pacientes, 74 (52,1%) homens e 68 (47,9%) mulheres, com média de 62 anos. A obesidade foi identificada em 16,2% dos pacientes. Pólipos foram encontrados em 61 (42,9%), sendo em sua maioria menores do que 1 cm. Obesos tiveram probabilidade 1,56 vez maior de apresentar adenoma colorretal que pacientes com peso normal.

***Conclusão*:**

Este estudo, apesar de mostrar a maior presença de adenomas colorretais em indivíduos obesos, não mostrou diferença estatisticamente significativa na ocorrência de lesões pré-malignas.

## INTRODUCTION

Affecting approximately one million people a year, colorectal cancer (CRC) is one of the biggest health problems worldwide. It is a common affection in developed countries and its incidence has progressively increased in undeveloped countries^6^.

Adenomas, the most common benign neoplasms of the colon and rectum, are known to be premalignant lesions that precede, in 10 to 15 years, the CRC, and correspond to about 70% of all intestinal polyps^24^. Although only one in 100-200 adenomas will turn out to be malignant, all adenocarcinomas of the large intestine appear in a dysplastic epithelium. Based on this theory, it is reasonable to consider that adenomas and carcinomas must have similar epidemiological characteristics and share a common etiology.

In view of the increasing prevalence of obesity^17^ ^,27^ and the high incidence and mortality of CRC, the objective of this study was to evaluate the association between obesity and the development of adenoma, a precursor lesion for colorectal cancer, since the accumulation of excess fat consists of a risk factor potentially modifiable through prevention strategies.

## METHODS

This study was carried out in accordance with the recommendations of the Declaration of Helsinki and Resolution no. 196/96 of the Ministry of Health on research involving human beings and approved by the Institution’s Ethics Committee number 79599917.7.0000.5336. To meet the objectives of the investigation, a cross-sectional study with consecutive patients of a qualitative and quantitative nature was adopted.

### Patients

The study population consisted of patients who sought the endoscopy service of a tertiary referral hospital in order to perform colonoscopy. The sample was represented by 150 individuals of both genders who authorized the use of their data and signed the free and informed consent form, aged between 18 and 90 years, submitted to the examination from December 2017 to December 2018. The inclusion criterion was to perform a complete examination, that is, visualization of the large intestine to the cecum, with adequate preparation of the colon. Patients who repeated the exam during the study interval, incomplete exam or poor colonic preparation were excluded.

### Anthropometric procedures

Weight was measured on a properly calibrated scale with a maximum capacity of 150 kg and subdivision at 100 g. Height was determined using a vertical millimeter anthropometer with a 0.5 cm scale. To measure the abdominal circumference, the patient was placed upright, with the abdomen relaxed, arms extended and weight equally distributed between the legs, with the feet close and parallel, and the measurement performed at the end of expiration, using a flexible and inelastic measuring tape, horizontally around the waist, at the midpoint between the last rib and the iliac crest.

The BMI calculation was performed using the formula that relates weight, in kg, to the height squared (m^2^), being adopted as a cutoff bridge to assess the nutritional status of those recommended by WHO.

### Endoscopic procedures

The preparation for colonoscopy included a liquid diet without residues and two pills of bisacodyl laxative 5 mg (Dulcolax^®^, Boehringer Ingelheim, São Paulo) on the eve of the exam; 1000 ml of 10% mannitol was prescribed to be ingested in 2 h on the day of the examination, and the consumption of clear liquids or water was released during the preparation, up to 2 h before the beginning of the examination.

Colonoscopies were performed by a single team and performed from the anal canal to the cecum or terminal ileum. Sedation was performed in all patients by an anesthesiologist. All lesions eventually found were characterized in terms of location and size, and removed by endoscopic polypectomy, with diathermic loop and cut-type electrical current. Flat, deep or larger than 25 mm lesions were only biopsied. After the examination was completed, all patients remained in the post-anesthetic recovery room, at rest, under medical and nursing observation. Then, they were released to return home, under responsible supervision, with medical guidance and contact phones for cases of complications.

### Statistical analysis

Descriptive analyzes were made of the categorical variables used in the study, with the elaboration of frequency distribution tables. Regarding the factors associated with the events, an independence study was carried out between the variables of presence of polyps with those that indicate obesity, both by BMI range and by waist circumference range (visceral obesity). For such tests, Pearson’s chi-square test was used at a significance level of 5%. That is, to accept the hypothesis of dependent variables (obesity being a risk factor for CRC), the level of statistical significance was set at p-value less than or equal to 0.05.

## RESULTS

A total of 150 patients who agreed to participate in the study were evaluated, but eight were excluded because they had incomplete examination and/or poor colonic preparation, resulting in 142 patients, of whom 74 (52.1%) were men and 68 (47.9%) women, aged 22-85 (61±11.05) years (Table 1). Colonic preparation was appropriate in all patients, allowing the colonoscopic examination to be performed satisfactorily, with progression of the device to the cecum or terminal ileum and adequate observation of the entire large intestine. In this series, there were no complications in relation to colonoscopy or the anesthetic procedure.

Regarding the body mass index, 38% of the patients had a normal BMI (18.5 to 24.9 kg/m^2^) and 16.2% were considered obese according to the BMI International Classification of Obesity. Considering the cut-off point of the WHO abdominal circumference of 94 cm in men and 80 cm in women, 124 patients with visceral obesity were identified, 93.2% men and 80.2% women.

Polyps were found in 61 patients, which is equivalent to a prevalence of 42.9% in this series, and most of them smaller than 1 cm. Polyps larger than 1 cm were found in 19.7% of the 61 patients who had them most frequently in the right colon. Adenomas were found in 38.7% of the 142 individuals who underwent the examination. Another frequent finding, diverticular disease, was observed in 35 patients (24.5%).

The factors associated with the occurrence of colorectal adenomas from the univariate analysis are shown in Table 2. By the analysis, men were 1.1 times more likely to have adenomatous polyp than women. Above 50 years of age, there was a 2.05 times greater probability of colorectal adenomas than below that age. Individuals considered obese from BMI (BMI >30.0 kg/m^2^) were 1.29 times more likely to have adenomatous colonic polyp than patients with normal weight. If we consider the increase in abdominal circumference as a risk factor for the occurrence of pre-malignant lesions, individuals with central obesity were 1.26 times more likely to have adenomatous polyps than individuals without visceral obesity. However, none of these factors were statistically associated with the occurrence of adenoma (p>0.05).

**TABLE 1:** Frequency distribution of the variables gender, age, body mass and waist circumference in individuals undergoing colonoscopy (n=142)

Variables	Frequency	Percentage
Gender Male Female	74 68	52.1% 47.9%
Age <50 y 50-60 y ≥60 y	15 46 81	10.6% 32.4% 57.0%
BMI Thin (<18.5 kg/m^2^) Normal (18.5 a 24.9 kg/m^2^) Overweight (25.0 a 29.9 kg/m^2^) Obesity (≥30.0 kg/m^2^)	02 54 63 23	1.4% 38.0% 44.4% 16.2%
Abdominal circumference Men <94 cm ≥94 cm Women <80 cm ≥80 cm	5 69 13 55	6.7% 93.2% 19.1% 80.9%

**TABLE 2:** Univariate analysis of the association of the variables gender, age and body mass index (BMI) with the occurrence of colorectal adenomas in individuals undergoing colonoscopy (n=142)

Variables	Without adenoma [n=87]	With adenoma [n=55]	RR [IC 95%]	p*
Gender Female *Ma*le	43 44	25 30	1.0 1.10 [0.73-1.67]	0.645
Age *<*50 y *>*50 y	12 75	3 52	1.00 2.05 [0.73-5.73]	0.262
BMI Non-obese (<30 kg/m^2^) *Obes*e (>30 kg/m^2^)	75 12	44 11	1.00 1.29 [0.79-2.10]	0.328
Abdominal circumference Without obesity Visceral obesity	21 66	45 10	1.00 1.26 [0.72-2.19]	0.403

RR=relative risk; CI=confidence interval; *=Pearson’s Chi-Square test

## DISCUSSION

CRC remains a serious public health problem due to its high incidence, mortality and diagnostic trend in more advanced stages. This has motivated several discussions about its importance and the need to make the population aware of the disease and the screening methods.

Colonoscopy is considered the gold standard for the diagnosis of CRC, as well as for its prevention, since it allows endoscopic resection of pre-neoplastic lesions^9^. Therefore, it is seen as a preventable disease, since it usually develops from a benign and slow-growing precursor lesion, the adenomatous polyp, which can be diagnosed and resected in screening colonoscopies.

In this study, it was possible to perform the complete examination in 96% of the patients. This result is in agreement with the American studies that report a cecum intubation rate of 97% in colonoscopies^22^. This fact was possibly facilitated by performing the exam under anesthesia, as it reduces anxiety and discomfort, and consequently improves the tolerability of the procedure and provides better conditions for the exam^7^. According to data from the American Society for Gastrointestinal Endoscopy, the morbidity of the procedure varies from 0.2% in diagnostic colonoscopies to 1.2% in therapies, with a mortality of 0.0006%^8^. In this case series, complications related to the anesthetic act were not verified and the exams were performed without complications.

In 142 studied patients, BMI between 18.43 and 43.29 kg/m^2^, mean of 26.61±4.15, 23 obese individuals were found, that is, 16.2% of the sample, thus labeled as having BMI >30 kg/m^2^. This data is in accordance with the incidence of obesity in Brazil, which, according to an estimate by the Ministry of Health, is 18.9%^5^.

Regarding the accumulation of fat in the abdominal region, according to the measures established by the WHO^2^, 124 patients with visceral obesity were found, which constitutes a risk factor for metabolic and cardiovascular diseases, even if the BMI is within the limits of normality^10^. Studies have pointed to abdominal circumference as an anthropometric measure best correlated to the amount of visceral adipose tissue, which is therefore associated with chronic non-communicable diseases^18^. They also highlighted the urgent need for the measurement of waist circumference to be adopted as a routine for the clinical history of patients, due to the acceptability of this measure by the population, practicality, simplicity and ease of interpretation. However, there is a scarcity of studies measuring abdominal obesity in individuals of normal weight and little is known whether the risk factors for overweight and abdominal obesity are the same.

With regard to colonoscopic results, most of the patients studied had a normal exam (57.04%). The most frequent diagnosis was polyp, found on 61 (42.9%) occasions, followed by diverticular disease on 35 (24.6%). A similar result was obtained in a prospective study that analyzed 9,223 colonoscopies in the United Kingdom, in which polyps were the most common findings, found in 22.5% of cases^4^. Adequate detection and treatment is of great importance, considering that most CRCs result from pre-existing adenomatous polyps (adenoma-adenocarcinoma sequence).

Among the individuals studied, 55 patients (39.4%) were diagnosed with colorectal adenoma. Adenomatous lesions, which present as polyps or flat lesions, are the neoplasms most frequently found in screening colonoscopies, as well as in symptomatic patients over 50 years of age^25^. Its incidence was higher than that found in the literature, which reports that ¼ of the patients have pre-malignant lesions. Necropsy studies have shown a prevalence of 20-30% for adenomatous polyps and report an increase in this incidence with age^20^.

In our study, the occurrence of colorectal adenoma, a precursor lesion for CRC, was higher in men (54.5%). A similar proportion in this incidence was found in a study in 2014, where 50.8% women and 58.2% men^12^ had the disease. This fact was attributed to the lifestyle of men, with greater exposure to risk factors such as smoking, excess body weight, high consumption of alcohol and red meat, low intake of fruits, vegetables and fibers, and physical inactivity^12^ ^,^ ^16^.

The presence of adenoma was greater in the age group older than 50 years; however, current studies demonstrate that, curiously, the incidence of CRC is increasing among young adults^3^. For this reason, American Cancer Society recently published new guidelines for CRC screening, recommending beginning screening for adults at age 45^1^.

In relation to the presence of pre-malignant lesions in obese patients, the proportion was higher than in individuals of normal weight or overweight, since 47.8% of this population had adenomatous lesions. Studies indicate that obesity is associated with an increase in the prevalence of benign colorectal neoplasia and, consequently, of CRC^13^ ^,^ ^15^ ^,^ ^23^. A meta-analysis conducted in westerners showed that the increase in BMI by 5 kg/m^2^ increased the risk of colorectal adenomas by 1.19 times, and also by CRC by 1.13 and 1.06 times in men and women, respectively^19^.

Regarding visceral obesity, although without results with statistical significance, our study is also in accordance with the literature, as it suggests that central obesity, represented by waist circumference, is a predictor of adenoma, regardless of BMI^11^ ^,^ ^14^.

There are some limitations to this study. It is worth noting an important limitation of colonoscopy, which are the unnoticed lesions, even in complete exams up to the cecum. Studies have found an average rate of undetected adenomas of 24% in general, 27% for adenomas smaller than 5 mm, 13% for adenomas between 6-9 mm and 6% for adenomas of at least 10 mm^21^. Such data show that although colonoscopy is considered the gold standard, it is a method that contains flaws. It is possible that our study was directed to those interested in their health, since it was made up of individuals who sought the endoscopy service in order to schedule the exam, which means that this sample may not represent the entire population. Another point to be emphasized is that most individuals in this series had a high socio-cultural level, and had been clarified about the need and importance of colonoscopy for the screening of CRC in routine consultations. We believe that for these reasons we did not find injuries in advanced stages and CRC during the study. The highest incidence of polyps with high-grade dysplasia/carcinoma in situ and even CRC occurs in individuals with less access to health and information resources, since they are not submitted to screening for this neoplasia and colonoscopy occurs in the presence of clinical manifestations of disease. In addition, extrapolation of these data should be done with caution, since it has limitations. First, we did not have data on physical activities and eating habits, which are risk factors constantly associated with the prevalence of adenoma and CRC. We also do not have information on the causality of obesity in the occurrence of colorectal neoplasms, since a cross-sectional design was used. It would be necessary to conduct large-scale studies, using a variety of obesity markers, and also to assess whether the treatment of obesity, whether through lifestyle changes, medications or operations, could lead to a decrease in the incidence of colorectal neoplasia.

## CONCLUSION

Both individuals with obesity estimated by BMI and those with visceral obesity were more likely to have adenomatous polyps, precursors to CRC; however, the results were not statistically significant with respect to malignancy.
